# Understanding amblyopia from the perspective of neurovascular units: changes in the retina and brain

**DOI:** 10.3389/fcell.2025.1590009

**Published:** 2025-06-27

**Authors:** Lin Zhang, Yaqi Zhao, Xiaolu Shi, Fan Wu, Yi Shen

**Affiliations:** ^1^ Special Education College, Beijing Union University, Beijing, China; ^2^ Experimental Research Center, China Academy of Chinese Medical Sciences, Beijing, China

**Keywords:** amblyopia, neurovascular unit, retina, brain, neural plasticity

## Abstract

As a developmental vision disorder, amblyopia has traditionally been studied with a focus on neurons. However, the neurovascular unit (NVU), a dynamic functional complex of neurons, blood vessels, and glial cells, has recently been implicated in amblyopia. This review systematically discusses the pathological changes and functional interactions of the NVU in retina and brain in amblyopia patients and experimental models, providing a new perspective for clinical intervention.

## Graphical Abstract

**Figure:**
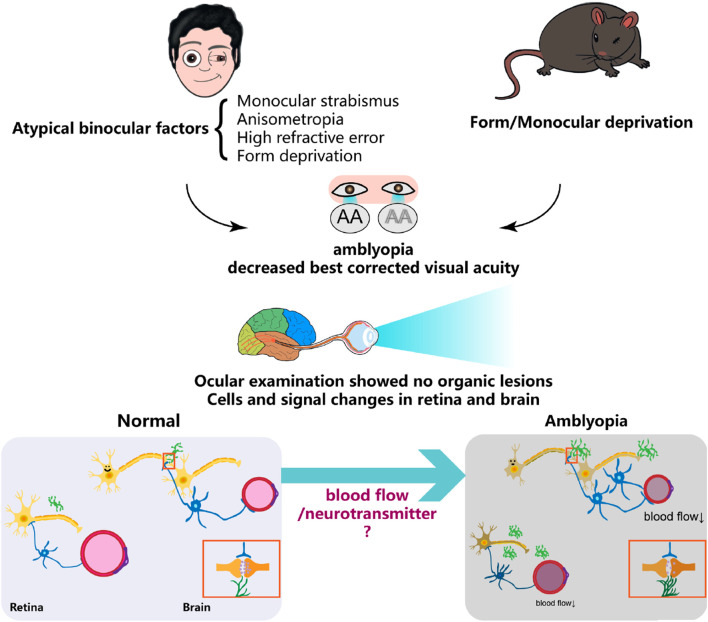
Summary diagram illustrating the formation of amblyopia, highlighting alterations in various cellular components of the neurovascular unit in the retina and brain, where blood flow and neurotransmitter regulation play a crucial role.

## 1 Introduction

Amblyopia is a condition characterized by reduced vision in one or both eyes due to abnormal visual experiences, such as vision deprivation, anisometropia, or strabismus, during the critical period of visual development. Amblyopia affects 2%–4% of children in North America and is the most prevalent cause of unilateral visual loss in children (Solebo and Rahi, 2014). A study involving 47,571 children identified refractive and strabismic amblyopia as the primary causes of visual impairment in this population (Kim et al., 2024). Furthermore, amblyopia is recognized as the leading cause of monocular visual impairment in adults (Tuna et al., 2024).

For decades, research into the pathological mechanism of amblyopia focused primarily on neural plasticity. Neural plasticity in amblyopia refers to the visual cortex’s ability to modify neural connections in response to abnormal visual input during the critical developmental period and beyond, thereby influencing visual function (Birch and Duffy, 2024; Sengpiel, 2014; Hensch, 2005). Neurovascular unit, a dynamic functional complex composed of neurons, vascular endothelial cells, pericytes and glial cells in amblyopia has been gradually revealed, that plays a vital role in both retina and brain (Grimes et al., 2024; Schaeffer and Iadecola, 2021; Metea and Newman, 2007). Associated with critical physiological processes, including the regulation of blood flow, metabolic support, and signal transduction, NVU influence neural plasticity and visual function, which was discovered in various ocular and neurological diseases (Jones et al., 2012; Wareham and Calkins 2020; Sipe et al., 2021). This review elucidates the functional and morphological alterations of NVU in the retina and brain under amblyopic conditions, as well as the related molecules, with the aim of identifying new targets for intervention. It is noteworthy that in discussing the pathological changes associated with the form deprivation model, this article addresses both amblyopia and myopia, as both conditions involve unilateral visual deprivation. Although myopia induced by form deprivation typically results in refractive errors through the application of diffuser lenses to a single eye, it may still offer valuable insights in the context of amblyopia research, particularly given the current limitations in amblyopia-related studies. Accordingly, the form deprivation myopia model may serve as a useful reference and source of novel perspectives.

## 2 Retinal NVU in amblyopia

Structural and functional alterations in the retina have been observed in amblyopia (Mompart-Martínez et al., 2023; Miller et al., 2020). Optical coherence tomography (OCT) and its variants have been widely used to assess retinal layer thickness in amblyopic eyes *in vivo*; however, the findings remain controversial (Gaier et al., 2019). Some studies report increased overall retinal thickness, particularly in specific layers such as the outer nuclear layer (ONL), the foveal and macular regions, and the retinal nerve fiber layer (RNFL) (Alqudah et al., 2025; Manouchehri et al., 2025; Li et al., 2015; Szigeti et al., 2014). Conversely, other studies have found no significant differences or even reduced retinal thickness, potentially due to variations in amblyopia subtypes (Parikh et al., 2022; Araki et al., 2017): For example, in high myopic amblyopia, retinal thickness is decreased (Wan et al., 2022), while in esotropic amblyopia, foveal and RNFL thickness remain unchanged (Xu et al., 2013). Moreover, in strabismic amblyopia, the ganglion cell complex (GCC) thickness is reduced (Tugcu et al., 2013). Experimental models further highlight these inconsistencies. In adult rodent models of amblyopia, the combined thickness of the RNFL, ganglion cell layer (GCL), and inner plexiform layer (IPL) has been reported to increase (Martinez-Navarrete et al., 2024). In contrast, form-deprivation models have shown overall retinal thinning, particularly in the RNFL, inner nuclear layer (INL), and ONL (Duan et al., 2023; Chen et al., 2020). These discrepancies may arise from differences in amblyopia modeling techniques, or more in depth, the distinct mechanisms of visual deprivation. While form-deprivation amblyopia often leads to retinal thinning due to early and severe stimulus loss, anisometropic amblyopia may induce compensatory thickening in certain layers. Methodological variations and neuroplastic responses may also contribute to these divergent findings. The most commonly used experimental model, monocular deprivation (MD), also referred to as monocular form deprivation (FD), is induced by covering one eye, which is thought to be the most severe type in amblyopia (Attebo et al., 1998). The method of occlusion significantly influences ocular outcomes. For instance, using a facemask to cover one eye in guinea pigs induces coexisting myopia and amblyopia (Tian et al., 2021), whereas eyelid suture deprivation has been associated with corneal flattening, a feature absent in models using opaque goggles (Norton, 1990). Monocular deprivation, achieved by blocking visual input to one eye, represents an extreme form of biased visual experience. It induces profound anatomical and physiological alterations in the primary visual cortex (V1), particularly in the cortical territory corresponding to the deprived eye, ultimately resulting in vision loss resembling human deprivation amblyopia (Mitchell and Sengpiel, 2009). Therefore, a systematic review of experimental animal models of amblyopia is essential to establish a scientifically robust foundation for clinical translation. Table 1 (Comparative Analysis of Animal Models for Amblyopia) aims to address key issues, including interspecies differences, the validity and limitations of specific models, and the critical importance of appropriate model selection in preclinical research (Duffy et al., 2023; Tian L. et al., 2021; Kiorpes, 2019; Mower, et al., 1982). Reliance on a single species--particularly rodents such as mice or rats, which possess relatively primitive visual systems, may limit the translational relevance of findings and fail to provide reliable guidance for the treatment of amblyopia in humans. In summary, the selection of animal species for amblyopia research should be tailored to the specific scientific goals and methodological requirements of the study. Non-human primates are the most suitable models for investigating strabismic and anisometropic amblyopia with high translational relevance, owing to their close anatomical and functional resemblance to the human visual system. In contrast, mice and rats are optimal for mechanistic, molecular, and genetically tractable investigations, especially where high-throughput or long-term experiments are needed. Cats and guinea pigs provide a valuable intermediate model, offering relatively high similarity to human binocular vision and are well-suited for functional and behavioral studies of amblyopia. Ultimately, experimental design should be guided by a comprehensive evaluation of the advantages, limitations, ethical constraints, and relevance to the research question, to ensure scientific validity and reproducibility.

**TABLE 1:** Comparative analysis of animal models for amblyopia.

Species	Modeling methods (with pros and cons)	Species-specific advantages	Species-specific limitations	Similarity to human
Mouse	1. Monocular deprivation via eyelid suture or face mask: fully blocks form vision, simple surgery, minimal distress. Mimics congenital cataract or ptosis 2. Anisometropia via contact lens: induces blur/defocus in one eye, lens difficult to secure, simulates anisometropic amblyopia	1. Low cost 2. Genetically tractable 3. Rapid development 4. Suitable for molecular studies	1. No fovea 2. Simplified cortex 3. Rudimentary visual system 4. Limited glial complexity	★★☆☆☆
Rat	Same as mouse	1. Suitable for behavioral testing 2. Axial length measurable	Same as mouse	★★☆☆☆
Guinea pig	Same as above, but monocular deprivation leads to co-existing myopia and amblyopia. Useful for studying overlapping mechanisms and treatment windows	1. Large eyes 2. High refractive plasticity 3. Complete cone system	1. Retinal/cortical divergence 2. Low visual acuity 3. Limited genetic tools	★★☆☆☆
Cat	1, 2 Same as above 3. Strabismus model via medial rectus transection induces image misalignment. Mimics congenital/early strabismus. Natural anisometropic and strabismic amblyopia possible	1. Human-like binocular system 2. Ideal for physiological recordings	1. High cost 2. Incomplete fovea 3. Surgical risk	★★★★☆
Tree shrew	Same as mouse	1. Fovea-like specialization 2. Developed visual cortex	1.Rarely used 2.Few tools/standards	★★★☆☆
Primates (e.g., acaques)	Same as cat	1. Closest human match 2. Has fovea 3. Eye movement and perceptual tasks 4. Homologous cortical/NVU structure	1. Very high cost 2. Long cycles 3. Limited access	★★★★★

To advance amblyopia research and enhance its clinical translatability, future animal studies should more systematically integrate ethical, biological, and methodological considerations into their design. Adherence to the 3R principles, Replacement, Reduction, and Refinement, remains a fundamental requirement to ensure both ethical responsibility and scientific rigor. Prior to conducting *in vivo* experiments, researchers should fully consult existing *in vitro* and computational data, optimize statistical design, and adopt non-invasive or minimally invasive procedures to minimize animal stress. Species selection should be tailored to the specific subtype of amblyopia and the intended depth of investigation. While rodent models play a pivotal role in elucidating molecular mechanisms, they present notable limitations in replicating the complexity of the human visual system, particularly in aspects related to glial architecture and neurovascular coupling. For studies requiring in-depth validation of mechanisms or therapeutic efficacy, cats and non-human primates, with brain structures more comparable to humans, may serve as valuable complementary models for preclinical validation. Moreover, the timing of experimental intervention is critical. Animal models should align, as closely as possible, with the critical periods of visual plasticity in human development (e.g., early postnatal stages) to ensure biological relevance. Future research may benefit from integrating cross-species comparisons, longitudinal assessments, and intervention-response analyses, thereby strengthening the translational bridge between basic science and clinical application in amblyopia.

Although rare studies to date have specifically examined the direct relationship between retinal layer thickness and NVU function in amblyopia models, existing evidence of structural alterations suggests that this is a promising area for further investigation, and one of the key focuses of our work. The integration of OCT/OCTA imaging with molecular biology approaches may facilitate the elucidation of neurovascular mechanisms at the retinal level in amblyopia. Changes in retinal layer thickness have been shown to significantly influence NVU structure and function in other conditions, for example, diabetic retinopathy. Notably, thinning of the inner retinal layers—such as the ganglion cell layer (GCL) and retinal nerve fiber layer (RNFL)—has been clearly associated with impaired local neurovascular coupling, indicating that structural degeneration serves as an important basis for functional deficits (Pemp et al., 2024). Before that, we should review the distribution of neurovascular units in the retina, which may help to understand the pathological basis of amblyopia. Prior to this, it is essential to review the distribution of NVU within the retina, as this may provide important insights into the pathological underpinnings of amblyopia.

Neurovascular units are exclusively distributed in the inner layers of the retina. Due to their metabolic sensitivity, nerve cells, astrocytes, and pericytes rely heavily on proper vascular support (Provis, 2001). In the retina, photoreceptors receive essential nutrients from the choroid, while the remaining retinal layers are vascularized by the central retinal artery. Upon entering the eye, this artery branches to form a trilaminar vascular network, consisting of the superficial, intermediate, and deep vascular plexuses (SVP, IVP, and DVP, respectively). The SVP, located within the ganglion cell layer (GCL) and the optic fiber layer, comprises veins, arterioles, and capillaries (Kornfield and Newman, 2014; Snodderly et al., 1992). Retinal astrocytes are confined to the SVP, whereas Müller cells span nearly the entire retinal thickness, extending fine lateral processes into all synaptic and vascular layers. Microglia are dispersed throughout the inner retina; under pathological conditions, they become activated and migrate to other layers. Photoreceptor cells, including cones and rods, have their cell bodies localized in the outer nuclear layer (ONL) and outer plexiform layer (OPL), where they form synaptic connections with bipolar and horizontal cells. The cell bodies of bipolar cells reside in the inner nuclear layer (INL), while their axons extend into the inner plexiform layer (IPL), forming synaptic connections with the dendrites of ganglion cells. Ganglion cell bodies are located in the GCL, with their axons converging to form the retinal nerve fiber layer (RNFL), which transmits visual information to the optic nerve. Horizontal cell bodies are situated between the ONL and INL, with axons extending into the OPL, where they establish synaptic connections with the dendrites of bipolar cells, modulating signal integration in the retina. Non-elongated cells have their cell bodies in the INL, with protrusions extending into both the INL and the plexiform layer, forming synaptic connections with bipolar and ganglion cells (Masland, 2001).

The thickness of specific retinal regions and layers is believed to serve as an indicator of pathological changes to some extent. For instance, RNFL thickness can be used as a marker for assessing axonal loss and neuronal degeneration (Yuan et al., 2023). Similarly, increased central macular thickness is thought to reflect delayed or disrupted pruning of intraretinal synapses in amblyopia. Retinal functional alterations in amblyopia can be revealed using modern imaging modalities such as OCT-A, electrophysiological tests (visual evoked potentials (VEP) and electroretinography (ERG), and microperimetry. In children with functional amblyopia, OCT-A has demonstrated a significant reduction in macular capillary density, particularly within the foveal region, which negatively correlates with visual acuity and appears reversible following occlusion therapy (Errera et al., 2024). Electrophysiological assessments and microperimetry further indicate decreased photosensitivity in the central retina of amblyopic eyes, which is associated with structural changes such as thinning of the ganglion cell layer and alterations in choroidal thickness (Khubieva and Tarutta, 2022). Following amblyopia treatment, changes in retinal and choroidal thickness have also been observed, suggesting that some structural alterations may exhibit neuroplastic potential, although the underlying mechanisms remain unclear (Jing et al., 2022). However, additional evidence is needed to clarify the role of retinal thickness alterations in amblyopia and their impact on the neurovascular unit. This underscores the necessity for further investigations into the underlying mechanisms and functional implications of these structural changes.

### 2.1 Retinal vascular alterations

The traditional understanding of amblyopia primarily attributes its underlying mechanism to functional inhibition within the visual cortex. However, recent studies suggest that alterations in retinal blood flow may also contribute to its pathogenesis, though it remains unclear whether these vascular changes are a cause or a consequence of amblyopia. In other ophthalmic disorders, such as diabetic retinopathy (Maurissen et al., 2024), age-related macular degeneration (Gattoussi et al., 2019), and pathological myopia (Si et al., 2024), changes in retinal vasculature and blood flow have been shown to reflect underlying pathophysiological processes, underscoring the potential relevance of similar mechanisms in amblyopia. In individuals with amblyopia, retinal blood flow parameters are altered, characterized by reduced blood flow velocity and vascular narrowing, which may lead to retinal undernutrition and subsequently impair the quality of refractive imaging. Furthermore, amblyopia may contribute to structural abnormalities in the retinal vasculature, including decreased vessel density and changes in vessel diameter, potentially disrupting normal retinal function (Yang et al., 2023; Cinar et al., 2020; De Schweinitz, 1907). It is well established that reductions in local perfusion and microvascular density compromise the structural integrity and functional coupling of NVU, resulting in neuronal metabolic dysregulation, impaired signal transduction, and ultimately progressive functional decline or irreversible neurodegeneration. Such disruptions have been substantiated across multiple models of retinal pathology (Maurissen et al., 2024; Si et al., 2024; Gattoussi et al., 2019).

Children with monocular functional amblyopia exhibit reduced vascular density (VD) and perfusion density in the macular region, suggesting impaired retinal microcirculation that may contribute to the pathophysiology of amblyopia (Errera et al., 2024). Specifically, the deep parafoveal plexus VD is decreased in amblyopic eyes, whereas foveal VD remains unchanged (Manouchehri et al., 2025). Additionally, both macular and optic disc VD are reduced in children with anisometropic amblyopia (Li et al., 2025). Alterations in macular retinal VD have been correlated with changes in best-corrected visual acuity, which is significantly lower in amblyopic eyes, suggesting a potential link between microvascular abnormalities and visual function impairment (Sesma et al., 2024). Furthermore, amblyopic eyes exhibit significant reductions in retinal VD and skeleton length, indicating a strong association between retinal vascular alterations and the pathophysiological state of amblyopia. These findings underscore the potential role of retinal microvascular changes in the development and progression of amblyopia (Su et al., 2024).

However, alterations in retinal blood flow in amblyopic eyes do not necessarily indicate the presence of retinal ischemia. Therefore, investigating changes in the choroid and other contributing factors may provide further insights into the retinal state, aiding in a better understanding of the vascular and functional adaptations associated with amblyopia. As the primary source of blood supply to the retina, the choroid plays a crucial role in delivering oxygen and nutrients to the outer retinal layers through its extensive vascular network, thereby supporting the maintenance of normal retinal function (Campbell et al., 2017). In the MD model, increased choroidal blood perfusion has been shown to alleviate scleral hypoxia and reduce scleral α-smooth muscle actin (α-SMA) expression, suggesting a potential regulatory role of choroidal circulation in mitigating scleral remodeling and hypoxia-induced changes (Zhou et al., 2021). In a guinea pig model of form-deprivation myopia (FDM) and 532-nm laser treatment, choroidal neovascularization was observed along with upregulation of the hypoxia-inducible factor-1 (HIF-1)-vascular endothelial growth factor (VEGF) signaling pathway, indicating the presence of tissue ischemia and hypoxia. These findings suggest that hypoxia-driven vascular changes may contribute to the pathophysiology of MD (Liu et al., 2022). However, there is still lack of relevant evidence on whether there is significant ischemic and hypoxic changes in the retina of amblyopia eyes. Therefore, the changes of retina can only be inferred from the expression of some related factors. As a key regulator of hypoxic responses, HIF-1 can directly target VEGF to stimulate vascular growth (Saint-Geniez et al., 2006; Ozaki et al., 1999). Notably, in the choroid-retinal pigment epithelium (RPE) complex, increased VEGF-A expression and decreased HIF-1ɑ expression have been shown to inhibit the progression of FDM (Che et al., 2025). Additionally, differentially acetylated proteins were significantly enriched in key metabolic pathways, including glycolysis/gluconeogenesis, the pentose phosphate pathway, retinol metabolism, and the HIF-1 signaling pathway. These findings suggest that post-translational modifications, such as acetylation, may play a crucial role in regulating metabolic and hypoxia-related processes in the retina. Several key metabolic enzymes, including hexokinase 2 (HK2), hexokinase domain-containing protein 1 (HKDC1), pyruvate kinase M (PKM), lactate dehydrogenase (LDH), glyceraldehyde 3-phosphate dehydrogenase (GAPDH), and enolase 1 (ENO1), exhibited decreased acetylation levels under FDM conditions. Altered lysine acetylation of these enzymes in the form-deprived retina may disrupt the dynamic metabolic balance of the retinal microenvironment by modulating enzymatic activity. These post-translational modifications could influence critical metabolic pathways, potentially contributing to the pathophysiological mechanisms underlying progression (Feng et al., 2023). What needs to be emphasized here is, form-deprivation amblyopia (FDA) and FDM share considerable similarities in terms of induction methods, developmental timing, and the deprivation of patterned visual input. Despite these parallels, their primary pathophysiological targets differ: FDA predominantly affects cortical plasticity and neural development, whereas FDM alters ocular growth and refractive regulation. Importantly, co-occurrence of both conditions has been demonstrated in animal models such as guinea pigs, suggesting potential mechanistic overlap. Given the more advanced understanding of FDM—particularly in retinal signaling, scleral remodeling, and dopaminergic modulation—these insights may provide a valuable framework for unraveling early-stage mechanisms in FDA. Future studies should therefore integrate perspectives from both fields to foster a more comprehensive understanding of visual development disorders.

The regulation of retinal blood flow and the maintenance of vascular homeostasis are essential for preserving normal retinal function. Proper blood circulation ensures an adequate supply of oxygen and nutrients, supporting cellular metabolism and overall retinal health. Similar to the blood-brain barrier (BBB), the blood-retinal barrier (BRB) plays a critical role in maintaining retinal homeostasis. However, under pathological conditions such as ischemia and inflammation, the integrity of the BRB may be compromised, leading to disruptions in NVU cell communication and microvascular structural damage. This breakdown exacerbates inflammatory responses, ischemic injury, and disease progression, further impairing retinal function (Antonetti et al., 2012; Huang et al., 2011). In diabetes mellitus-induced retinal vascular dysfunction, the crosstalk between vascular pericytes and endothelial cells plays a crucial role in microvascular stabilization and remodeling. Disruptions in this interaction can lead to pericyte loss, endothelial dysfunction, and increased vascular permeability, thereby contributing to the progression of diabetic retinopathy and retinal microvascular complications (Liu et al., 2019). Pericytes, as multifunctional cells with plasticity and regenerative potential, are critical components of the NVU in both the brain and retina. Their interactions with endothelial cells contribute to vascular stability, permeability regulation, and tissue homeostasis, underscoring their essential role in neurovascular integrity (Huang, 2020). Despite the recognized importance of pericytes in retinal vascular function, there are only a few early studies examining their role in composite models combining MD and diabetes. Preliminary findings suggest that such models exhibit reduced pericyte loss and thickening of the capillary basement membrane (Zhang et al., 2024). Therefore, future research should focus on elucidating the specific changes in retinal pericytes and endothelial cells in amblyopia. Investigating their interactions, structural alterations, and functional dynamics will provide deeper insights into the role of microvascular dysfunction in amblyopia pathophysiology and may contribute to the development of targeted therapeutic strategies.

Emerging evidence suggests that retinal microvascular alterations, characterized by reduced perfusion, vessel density, and NVU disruption, may contribute significantly to the pathophysiology of amblyopia, paralleling mechanisms observed in other retinal disorders. Given the structural and functional overlap with form-deprivation myopia, insights from well-characterized myopia models may provide a valuable framework for advancing our understanding of amblyopia’s early-stage vascular and metabolic dysregulation.

### 2.2 Retinal glia alterations

The human retina comprises three types of glial cells: microglia and two types of macroglia, astrocytes and Müller cells. Macroglia play a crucial role in maintaining homeostasis and providing metabolic support to photoreceptors and neurons, which is essential for proper neuronal function (Reichenbach and Bringmann, 2020).

Retinal astrocytes and Müller cells establish direct contact with blood vessels and exhibit increased activation and elevated protein expression in a mouse FDM model. In this model, glial fibrillary acidic protein (GFAP)-positive structures adopt a more star-like morphology with enhanced connections to retinal vasculature, suggesting that hypoxia influences hormone metabolism, peptide secretion, and cellular differentiation (Zhang et al., 2022). Hypertrophied Müller cells are indicative of retinal distress and have been associated with worsened visual deficits, as observed in zebrafish with lazy eyes mutations, where Müller cell dysfunction compromises photoreceptor function and contributes to partial blindness (Kainz et al., 2003). In FDM in rabbits, Müller cells exhibit significant metabolic alterations, particularly in glycolysis and angiogenesis. The upregulation of glycolytic enzymes, including lactate dehydrogenase A and pyruvate kinase, suggests an adaptive response to increased metabolic demands under FD stress (Moon et al., 2024).

Microglial activation is observed, accompanied by migration toward the ganglion cell layer (GCL) and subsequent cell apoptosis in MD (Ma et al., 2020). However, the role of retinal microglia in amblyopia remains insufficiently explored, highlighting the need for further investigation into their activation patterns, functional alterations, and potential contributions to amblyopic pathophysiology. However, the changes observed in microglia under ischemic and hypoxic conditions may offer valuable insights. Oxygen-induced retinopathy (OIR) is a well-established model for retinopathy of prematurity, and both conditions share similar pathological processes. In OIR retinas, the number of microglia increases significantly, with most exhibiting an activated state. These activated microglia closely associate with newly formed blood vessels. Notably, microglial depletion via clodronate liposomes and inhibition of microglial activation with minocycline both lead to a reduction in pathological neovascularization (Xu et al., 2018). In OIR, activated microglia secrete various inflammatory cytokines, including Tumor necrosis factor-alpha (TNF-α), Interleukin-1 beta (IL-1β), and Interleukin-6 (IL-6), contributing to the inflammatory environment (Li et al., 2021). Additionally, microglia release proangiogenic factors such as fibroblast growth factor 2 (FGF2) and vascular endothelial growth factor A (VEGFA), which directly promote angiogenesis (Blank et al., 2021). As it is known, microglia activate and migrate in response to inflammatory mediators TNF and other stimuli, thereby exacerbating retinal lesions (Lennikov et al., 2019). Under hypoxic conditions, activated microglia promote the formation of new retinal blood vessels and contribute to vascular lesions, ultimately leading to increased retinal ganglion cell apoptosis (Liu et al., 2021; Yin et al., 2017).

These findings underscore the pivotal roles of retinal glial cells, particularly astrocytes, Müller cells, and microglia, in mediating neurovascular responses under stress conditions such as form deprivation and hypoxia. Macroglia demonstrate metabolic and structural plasticity in response to altered retinal demands, while microglia act as key modulators of inflammation and angiogenesis, potentially amplifying retinal damage. Although evidence from FDM and OIR models provides valuable insights, the specific contributions of glial cells to the pathophysiology of amblyopia remain insufficiently characterized. Therefore, further investigation into glial dynamics is essential and may reveal critical therapeutic targets for visual impairments associated with amblyopia and other retinal disorders. To investigate glial cell alterations in the amblyopic retina, advanced techniques such as tissue clearing combined with high-resolution imaging, single-cell metabolomics, and spatial metabolomics may offer precise insights into specific glial subpopulations. When integrated with the expression profiles of key cellular factors and signaling pathways discussed above, these approaches could contribute to the construction of a comprehensive and fine-grained retinal atlas, ultimately advancing our understanding of the cellular and molecular landscape underlying amblyopia.

### 2.3 Retinal neural cells changes

Over the past several decades, amblyopia has primarily been regarded as a cortical disorder; however, accumulating evidence suggests that detectable structural and functional changes also occur at the retinal level, particularly in form-deprivation and anisometropic amblyopia models (Mitchell and Sengpiel, 2009). Although retinal neurons are not the primary site of pathology, retinal ganglion cells (RGCs) and bipolar cells have been shown to undergo measurable synaptic, functional, and structural alterations. Visual deprivation induces changes at functional, cellular, and molecular levels within the retina. Specifically, in the deprived eye, RGCs exhibit an increased density and multilayered distribution. At the molecular level, several transcripts associated with retinal differentiation, such as fibroblast growth factor 2 (FGF-2), show altered expression patterns (Prokosch-Willing et al., 2015). Emerging technologies—including OCT, electroretinography (ERG), spatial metabolomics, and high-resolution *in vivo* imaging—offer promising tools to further map and characterize the neural circuitry alterations within the amblyopic retina.

In amblyopia models, retinal neurons undergo not only functional suppression due to visual input deprivation but also structural and molecular remodeling. Form deprivation, by eliminating patterned input to photoreceptors, initiates a cascade of changes that permanently alter neuronal architecture, disrupt synaptic connectivity, and modify pharmacological response profiles within the retina (Jones et al., 2012). At the molecular level, the sorting protein-related receptor SorCS1, a transmembrane protein involved in neuronal development, synaptic signaling, and metabolic regulation, is significantly upregulated in the retinas of FD rats. SorCS1 overexpression has been shown to enhance neurite outgrowth, promote cell viability, and increase the expression of inhibitory neurotransmitters (Chen et al., 2020). Additionally, disturbances in Ca^2+^-dependent signaling have been implicated in reduced neuronal activity within amblyopic retinas (Li et al., 2022). In primate models, altered visual experience caused by lid fusion increases mitotic activity in neurogenic progenitor cells within the peripheral retina, indicating that neurogenesis in the developing retina is modulated by visual input. Vasoactive intestinal peptide (VIP) has been identified as a key molecular mediator that promotes this activity and supports retinal growth (Tkatchenko et al., 2006). VIP’s role extends beyond the retina; it is widely expressed in the cerebral cortex, intraocular tissues, and the lateral geniculate nucleus (LGN) (Ogawa-Meguro et al., 1992; Fuxe et al., 1977; Said and Rosenberg, 1976). Exogenous VIP administration has been shown to suppress the pathological progression of form deprivation, improve neurochemical metabolism in the LGN, and facilitate visual recovery during the critical period of amblyopia (Li et al., 2019; Cakmak et al., 2017). Furthermore, VIP-positive interneurons play a pivotal role in cortical plasticity. These cells co-release VIP and the inhibitory neurotransmitter GABA, and are implicated in regulating stimulus-specific enhancement (SSE). Notably, SSE persists even in the absence of VIP itself, but is abolished when GABA release from VIP-expressing interneurons is impaired—suggesting that Hebbian plasticity mechanisms underlie SSE in adult V1 (Kaneko et al., 2024).

These observations highlight that amblyopia involves not only transient functional suppression, but also long-lasting structural and molecular remodeling within the retina. Visual input plays a fundamental role in guiding neuronal development, influencing synaptic connectivity, neurogenesis, and neurotransmitter expression. Molecules such as SorCS1 and VIP emerge as key regulators of retinal and cortical plasticity, with VIP demonstrating a broad influence across retinal, thalamic, and cortical circuits. These findings underscore the intricate interplay between visual experience and neurochemical signaling in shaping retinal architecture and function, offering promising avenues for targeted interventions in amblyopia. Integrating advanced imaging, optogenetic, and transcriptomic techniques in primate and human-relevant models are supposed to translate these findings into clinically applicable strategies.

## 3 Pathological alterations of the NVU in the amblyopic brain

As mentioned above, amblyopia results in abnormal development of the visual region of brain and impaired visual function, necessitating an understanding of underlying changes at the transcriptional level. For instance, the mRNA sequencing of the dorsal lateral geniculate nucleus (dLGN), the important relay stations of visual pathway, in the model of amblyopia revealed enrichment of gene pathways related to synaptic function, nervous system development, and neurogenesis (Lin et al., 2022). This also once again confirms the vital position of neuroplasticity in amblyopia. Recently, research has progressively shifted from a neuron-centric approach toward examining the neurovascular unit, recognizing that neuronal function and activity are not isolated phenomena but involve complex, bidirectional interactions with glial cells, vascular endothelial cells, and pericytes. Consequently, modulating blood supply to the visual cortex represents a promising new therapeutic perspective. However, to fully appreciate this approach, it is essential first to review the classical pathological mechanisms underlying neuronal plasticity and amblyopia.

### 3.1 Neural plasticity

Disrupted visual input during the early postnatal “critical period” of heightened neuroplasticity induces enduring structural and functional abnormalities across the visual pathway, including the retina, lateral geniculate nucleus (LGN), and visual cortex (Thompson, 2021). Amblyopia subtypes, strabismic, anisometropic, deprivation, and combined, arise from distinct disruptions in early visual experience and may reflect differing temporal profiles of neural plasticity (Mitchell and Maurer, 2022; Olson and Freeman, 1980).

While amblyopia has traditionally been viewed as a cortical disorder, recent evidence reveals significant retinal and thalamic (LGN) contributions. Retinal ganglion cells (RGCs), bipolar neurons, and synaptic structures undergo remodeling in animal models of deprivation amblyopia (Lynne, 2019; Mitchell and Sengpiel, 2009). These changes involve altered neurotransmission, disrupted gene expression such as SorCS1 (Chen et al., 2020), FGF-2 (Prokosch-Willing et al., 2015), and Egr-1 (Wang et al., 2024), and glial interactions. In the LGN, MD leads to shifts in ocular dominance (Jaepel et al., 2017; Rompani et al., 2017; Sommeijer et al., 2017; Morgan et al., 2016; Hammer et al., 2015; Hess et al., 2009), reduced excitability (Duménieu et al., 2025), and diminished plasticity-related gene expression, suggesting the LGN is a site of active plasticity rather than a passive relay. Two primary mechanisms of neuroplasticity underlie amblyopia: Hebbian plasticity (activity-dependent synaptic strengthening) and homeostatic plasticity (activity normalization) (Thompson et al., 2024; Yee et al., 2017; Turrigiano, 2012; Mrsic-Flogel et al., 2007). Both mechanisms interact within the visual cortex and are modulated by local inhibitory circuits, especially those involving parvalbumin-positive (PV^+^) interneurons. Restoration of excitatory/inhibitory (E/I) balance via visual stimulation or genetic manipulation, such as REST knockdown (Shmal et al., 2024), can reactivate plasticity and promote recovery.

Therapeutic interventions aim to reopen plasticity windows in the visual cortex. Approaches include dark exposure (Stryker and Löwel, 2018), fluoxetine administration (Maya Vetencourt et al., 2008), enriched environments, physical activity, temporary retinal inactivation via intraocular tetrodotoxin (TTX) (Fong et al., 2016; Linden et al., 2009; Stryker and Harris, 1986), and electroacupuncture (Wang et al., 2025). These methods act by reducing cortical inhibition, modulating GABAergic tone (Venturino et al., 2021; Greifzu et al., 2014; Sale et al., 2007), reshaping NMDA receptor function (Hu et al., 2024; Fong et al., 2020; Murphy et al., 2004; Philpot et al., 2001; Quinlan et al., 1999), and altering extracellular matrix architecture (Murase et al., 2017). Notably, NMDA receptor expression appears unaffected in the LGN (Ziburkus et al., 2000). Contrary to earlier beliefs, adult visual cortex retains latent plasticity. Visual recovery in adult amblyopic models demonstrates that synaptic remodeling, neurogenesis, and molecular signaling (e.g., CREB/BDNF/TrkB pathway) continue beyond childhood. Persistent synaptic connectivity depends on active visual input; unused synapses retract, but functional input can restore them. Synapses influence visual function by regulating local excitation/inhibition balance (Huang et al., 2024).

The LGN and V1 should be viewed as dynamic plastic hubs. In amblyopia, dLGN neurons show reduced responsiveness (Duménieu et al., 2021; Rose and Bonhoeffer, 2018; Sherman, 2007), changes in ocular dominance, and molecular reprogramming (Wang et al., 2024). These findings challenge the classical view of the LGN as a passive relay and underscore its active role in visual plasticity (Ikeda and Wright, 1976; Wiesel and Hubel, 1963). Together, these findings underscore that amblyopia is a system-level disorder involving multilayered neuroplastic changes from the retina to the cortex. Future therapeutic strategies should harness both early and late-phase plasticity mechanisms to optimize visual recovery across age groups.

Neuroplasticity profoundly influences the NVU by modulating neuronal activity, glial function, and local vascular remodeling. The dynamic interactions ensure metabolic support, maintain homeostasis, and facilitate adaptive changes during development and in response to injury or deprivation. Understanding this cross-talk is essential for developing targeted therapies for visual and neurological disorders such as amblyopia.

### 3.2 Abnormal cerebral blood flow and neuron-vascular coupling

Associations between visual abnormalities and reduced cerebral blood flow, along with impaired brain region functionality, have been well documented and are believed to contribute to neurophysiological changes (Bennett et al., 2020; Choi et al., 2001). It is important to emphasize that changes in cerebral blood flow (CBF) are distinct from structural alterations in the vasculature. This distinction highlights the need to integrate both *in vivo* and *ex vivo* approaches when investigating vascular and hemodynamic changes. Neurovascular coupling (NVC) refers to the coordinated mechanism by which local CBF increases in response to neuronal activation in specific brain regions, ensuring an adequate supply of oxygen, glucose, and metabolic substrates. NVC is fundamental to maintaining neural function, interpreting brain imaging signals (e.g., fMRI), and understanding the pathophysiology of neurological disorders.

In patients with visual deprivation amblyopia (VDA), significant reductions in cerebral blood flow within V1 have been reported, accompanied by corresponding neurophysiological and histopathological alterations in both the LGN and V1 regions (Pescosolido et al., 2014; Li et al., 2012). Reduced glucose metabolism in the V1 and decreased activation in extrastriate regions have been observed in patients with amblyopia (Hoyt, 2005; Demer et al., 1988). Blood oxygen level-dependent functional magnetic resonance imaging (BOLD-fMRI) utilizes the focal uncoupling between cerebral blood flow and metabolism to detect brain activation patterns (Born et al., 1998; Sereno et al., 1995; Schneider et al., 1993; Blamire et al., 1992; Ogawa et al., 1992; Belliveau et al., 1991), and has been employed to assess cortical changes in various types of amblyopia. Using this technique, patients with amblyopia have demonstrated reduced activation levels and decreased activated areas within Brodmann area (BA) 17 and additional extrastriate cortical regions. Furthermore, patients with anisometropic amblyopia show particularly pronounced cortical impairment within bilateral extrastriate areas BA 18 and BA 19 compared to those with strabismic amblyopia (Wang et al., 2012).

These phenomena may be explained by the intricate functional connection between the eye and brain. Stress hormones released by the brain can influence vascular tone, particularly affecting blood vessels in and around the optic nerve, impairing vascular autoregulation and neural metabolism (Sabel et al., 2018). Consequently, investigating the relationship among neurovascular coupling, neurotransmitter balance, and functional disturbances within the visual cortex is crucial for understanding the neurophysiological mechanisms underlying amblyopia (Zhang et al., 2024).

5-HT, a crucial neurotransmitter and vasoactive substance, exerts multifaceted effects on cerebrovascular regulation and cerebral hemodynamics. Its impact varies depending on the specific 5-HT receptor subtype, the type of blood vessel involved, and the prevailing physiological or pathological conditions. Its receptor abnormalities significantly contribute to the neurophysiological alterations observed in amblyopia. Interactions between disrupted serotonin receptor systems and impaired cerebral metabolism result in compromised neurovascular coupling and altered cortical responses (Carhart-Harris and Nutt, 2017). Specifically, activation of 5-HT1a receptors inhibits long-term potentiation within the visual cortex, whereas activation of 5-HT1b receptors modulates cortical visual responses. This interaction suggests a critical role for serotonin receptors in visual cortical plasticity. Fluoxetine, a selective serotonin reuptake inhibitor, has been shown experimentally to restore plasticity and improve visual function in adult amblyopic animal models by modulating serotonin receptor signaling (Maya Vetencourt et al., 2008). However, clinical studies in humans have provided inconsistent results; fluoxetine treatment has not uniformly enhanced perceptual learning or visual function in adult amblyopia patients, suggesting variability in therapeutic efficacy and potential limitations in clinical translation (Sharif et al., 2019; Huttunen et al., 2018).

Dopamine (DA) serves not only as a crucial neurotransmitter in CNS but also plays a significant role in the cerebrovascular system. It actively regulates cerebrovascular tone, modulates blood flow distribution, and facilitates neurovascular coupling, thereby influencing cerebral hemodynamics and overall brain function (Choi et al., 2006). Because dopamine readily crosses the blood-brain barrier, its precursor levodopa has been extensively investigated in clinical studies for amblyopia treatment, especially since visual deprivation is known to reduce dopamine concentrations (Holmes et al., 2016; Pediatric Eye Disease Investigator Group, 2010; Repka et al., 2010; Dadeya et al., 2009; Leguire et al., 2002; Mohan et al., 2001). Dopamine deficiency has been linked to impaired visual processing and disrupted neurovascular coupling, particularly involving abnormalities within dopamine signaling pathways. Furthermore, modulation of inhibitory circuits in the visual cortex is known to depend on the dopaminergic system, specifically involving D2 receptors and dopamine transporter (DAT) activity. Despite promising experimental results, clinical consensus regarding the effectiveness of dopamine modulation in amblyopia treatment remains controversial, as visual improvements vary among patients and optimal therapeutic approaches are still debated.

Moreover, abnormal cortical neuronal coupling in amblyopia is associated with elevated activity of metabotropic glutamate receptors, leading to increased impulsivity and functional impairments within the dopaminergic signaling pathway. Additionally, developmental alterations in GABA_a_ receptor subunit composition during accelerated neocortical maturation enhance γ-aminobutyric acid (GABA)-mediated inhibitory transmission, contributing to reactive declines in visual acuity. The noradrenaline transporter also plays a critical role in modulating visual cortical activity, thus influencing cortical executive function. Importantly, neurovascular coupling disturbances in amblyopia interact closely with vulnerabilities in both the noradrenergic transporter and GABA_a_ neurotransmitter systems, further highlighting the complexity of neurotransmitter interactions underlying amblyopic visual dysfunction (Zhang et al., 2024).

In summary, amblyopia is not solely a disorder of neural activity but also involves long-term remodeling of the structure and function of the neurovascular system. The studies above suggest that MRI-based neuroimaging can be used to assess plastic changes in cerebral blood flow pathways and vascular architecture in amblyopic brains, offering a potential avenue for the development of novel neurovascular-targeted therapies. However, the morphological and functional alterations of vascular-associated cells, such as endothelial cells, smooth muscle cells, and pericytes, within pathological brain tissues remain largely unexplored. Current insights into changes in the vascular components of the neurovascular unit are mostly inferred indirectly through related neurotransmitter profiles and signaling pathways, which highlights a critical need for future experimental animal studies focusing on cellular-level vascular changes in amblyopia.

### 3.3 Glia activation

During postnatal development, the remodeling of cortical neural circuits is essential for adapting to sensory input. Amblyopia affects the visual pathway at multiple levels, including retinal input, integration within the lateral geniculate nucleus (LGN), cortical processing, and white matter structure and connectivity (Miller et al., 2020). Different subtypes of amblyopia influence distinct components of the pathway, with the ON pathway, magnocellular (M) pathway, and thalamo-cortical white matter projections being particularly vulnerable (Liu et al., 2020; Pons et al., 2019; Allen et al., 2015). Among these regions, the primary visual cortex (V1) is the earliest and most severely affected area, particularly in monocular deprivation models, where responses from the contralateral dominant eye in V1 are significantly reduced (Kiorpes, 2019). Disruption of NVC, the dynamic balance between cerebral blood flow and neuronal activity, may further constrain the development and plasticity of these circuits; however, the causal direction and temporal sequence of these effects remain unclear, which may be a promising target for future therapeutic intervention. Besides, neuronal activity is modulated by visual experience, while interactions with glial cells further shape the structural and functional architecture of cortical circuits.

Astrocytes play an important role in this process, regulating synaptic plasticity, neuronal maturation, and local blood flow (Stevens, 2008). Astrocytes have reactive and non reactive states, and their activities are significantly affected by visual experience. Long term visual deprivation (such as dark rearing) reduces GFAP (glial fibrillary acidic protein) expression, indicating a decrease in astrocyte reactivity (Onwordi et al., 2020). However, the specific functional status of astrocytes and their role in cortical development and neural plasticity still need to be further explored, especially in the context of amblyopia and other sensory disorders. In V1 (primary visual cortex), long-term visual deprivation leads to a decrease in the number of reactive astrocytes, but no change in the total number of astrocytes (Wang et al., 2022). Enhancing gap junction coupling between astrocytes may serve as a compensatory mechanism to stabilize local neural circuits damaged by sensory deprivation (Rouach et al., 2000). This adaptive change reflects the regulatory effect of neural activity on astrocyte neuron interaction, indicating that astrocytes not only passively respond to external stimuli, but also maintain the functional homeostasis of neurons through coupling enhancement.

Visual experience during development significantly shapes astrocytic structure; however, simply altering visually driven neural activity in adulthood does not induce substantial structural changes in astrocytes (Hawrylak and Greenough, 1995). Furthermore, astrocytic glutamate transporters, particularly GLT1, mediate critical roles in the refinement of eye-specific inputs in the developing visual cortex by coordinating experience-dependent neuronal signaling and glutamate clearance (Sipe et al., 2021). In visually deprived cortex, astrocyte density rapidly increases until maximal functional recovery is achieved. Functional modulation of protoplasmic astrocytes exerts long-lasting influences on the functional recovery of cortical neurons following sensory deprivation, potentially by altering neuronal thresholds for reactivation (Hennes et al., 2020). Although astrocytes undergo significant changes in response to visual deprivation, their overall developmental trajectory is not disrupted by sensory loss. Specifically, the maturation of GFAP expression occurs early during postnatal development and remains unaffected by visual deprivation (Corvetti et al., 2003).

Visual experience shapes cortical neural circuits but also modulates the functional state of astrocytes. Prolonged visual deprivation can alter astrocyte reactivity, enhance astrocytic coupling, and influence the synaptic microenvironment. These adaptations may serve as crucial mechanisms for maintaining cortical homeostasis and facilitating functional recovery. Future research should further investigate the role of astrocytes in amblyopia, sensory deprivation adaptation, and visual restoration to uncover their fundamental contributions to neural plasticity.

Visual information originating from each retina remains largely segregated until converging at the cortical level. Consequently, multiple brain regions exhibit altered activation in both amblyopia patients and animal models, including V1, early extrastriate visual areas (V2 and V3), dorsal visual pathway regions such as area V3A and the middle temporal (MT) area, ventral pathway regions including area V4, and the inferior temporal cortex, particularly the rostral lower bank of the superior temporal sulcus (STS). Additional areas activated include regions within the parietal cortex, notably the lateral bank of the intraparietal sulcus (IPS), caudal intraparietal area (CIP), lateral intraparietal area (LIP), anterior intraparietal area (AIP), and the frontal eye field (FEF), as well as the ventral premotor cortex (area F5), known to contain visuomotor neurons receiving projections from the AIP (Joly and Frankó, 2014). Given the well-established link between amblyopia and neuroplasticity, microglia-mediated removal of inhibitory synapses within these regions appears critical for cross-modal cortical plasticity. Microglia selectively phagocytose inhibitory inputs onto visual cortical neurons, facilitating heightened neuronal responsiveness during sensory stimulation, with matrix metalloproteinase 9 (MMP9) playing a pivotal role in this process (Hashimoto et al., 2023).

Microglia play a crucial role in synaptic pruning, maturation, functional connectivity, and plasticity during cortical development. The proper maturation and connectivity of cortical circuits rely heavily on microglial activity. Notably, microglial depletion significantly reduces dendritic spine pruning during the critical period of visual cortex (VC) development, underscoring their essential role in glutamatergic synapse formation, refinement, and the establishment of functional neural circuits in early brain development (Ma et al., 2020). In the visual cortex, microglia respond to monocular deprivation by increasing lysosomal activity, indicating their active involvement in experience-dependent synaptic remodeling. However, signaling through the fractalkine receptor CX3CR1 does not appear to be essential for visual cortical development or synaptic plasticity (Schecter et al., 2017). This suggests that microglial regulation of cortical plasticity may occur independently of direct neuronal communication via the CX3CR1 pathway. In contrast, the purinergic receptor P2Y12, which is selectively expressed in non-activated microglia, plays a critical role in microglial motility and early injury responses. Disruption of P2Y12 receptor signaling significantly impairs microglial responses to monocular deprivation and effectively abolishes ocular dominance plasticity, highlighting its indispensable role in microglia-mediated synaptic remodeling within the visual cortex (Sipe et al., 2016).

Unlike many other neurological conditions where oligodendrocytes primarily serve supportive roles, in amblyopia they undergo significant adaptive remodeling. Monocular deprivation induces selective, neuron class-specific changes in myelination, highlighting the specificity of neuron-oligodendrocyte interactions (Yang et al., 2020). Oligodendrocyte maturation occurs slowly and progressively stabilizes neuronal circuits, thus restricting plasticity as animals mature (Priebe and McGee, 2014; LeVay and Stryker, 1978; Hensch, 2005). Importantly, in adult mouse models lacking adolescent oligodendrogenesis, brief monocular deprivation significantly reduced inhibitory synaptic transmission. This finding underscores the critical role oligodendrocytes play in the maturation and stabilization of cortical circuits and supports the concept that developmental myelination functions as a physiological brake on neuronal plasticity (Xin et al., 2024).

In mice, long-term monocular deprivation increases oligodendrogenesis in the retinogeniculate pathway but leads to shorter myelin internodes without altering other structural properties of myelinated fibers. Glutamate signaling may mediate this activity-dependent regulation of myelin internode length. Visual deprivation-induced shortening of internodes significantly decreases nerve conduction velocity in the optic nerve, demonstrating that sensory input critically shapes myelination and thus dynamically modulates the conductive properties of neural circuits in response to environmental experience (Etxeberria et al., 2016). Moreover, the average length of myelin internodes formed by individual oligodendrocytes varies depending on the proportion of sensory-deprived versus normally active axons. A higher proportion of deprived axons around an oligodendrocyte significantly alters its myelination pattern, indicating that oligodendrocyte myelination programs are strongly influenced by the local axonal microenvironment (Osanai et al., 2018). Physiological Nogo receptor (NgR) signaling induced by myelin-associated inhibitors, such as Nogo, MAG, and OMgp, helps consolidate neural circuits established during experience-dependent plasticity. However, in pathological contexts such as monocular deprivation, similar NgR-mediated signaling pathways can limit functional recovery and restrict axonal regeneration, thereby constraining visual cortex plasticity (McGee et al., 2005).

Beyond neuronal dysfunction, amblyopia may profoundly reshape brain structure and function through glial mechanisms—including metabolic support, synaptic pruning, myelination, and neurovascular regulation. Despite glia’s essential role in visual cortical development and excitation-inhibition balance, their involvement in amblyopia remains underexplored. Future studies should employ techniques such as immunohistochemistry, single-cell RNA sequencing, and *in vivo* imaging to elucidate glial dynamics and their mechanistic contributions in amblyopic brains.

## 4 Other signals affecting amblyopia

In addition to 5-HT and DA discussed earlier, other neurotransmitters also contribute to the NVU in amblyopia. Citicoline, a complex organic molecule known for increasing norepinephrine and dopamine levels within the central nervous system, has been evaluated as a treatment in ocular disorders such as amblyopia and glaucoma due to its neuro-modulatory effects. It has been shown to compensate for the reduction in retinal dopamine levels and mitigate the pathological shift in FD guinea pigs (Campos et al., 1995). Studies have demonstrated that citicoline administration significantly improves visual acuity and contrast sensitivity, even showing therapeutic potential in both amblyopia children and adults beyond the critical period of visual system development (Pawar et al., 2014; Porciatti et al., 1998; Campos et al., 1995).

The neurotransmitter acetylcholine is known to facilitate plasticity in animal models by elevating cortical acetylcholine levels during passive visual exposure. In the posterior sclera, expression of the muscarinic acetylcholine receptors M1 and M4 subtypes significantly increased in FDM eyes (Liu et al., 2007). Donepezil, a cholinesterase inhibitor used to enhance cortical acetylcholine levels, has thus been investigated as a potential therapeutic strategy for amblyopia, while its effectiveness remains controversial. Donepezil significantly reduces the magnitude and duration of the shift in the process of a-few-hour MD (Sheynin et al., 2019). As the limited efficacy indicating, donepezil did not improve perceptual learning in adults with amblyopia and, in some cases, was associated with impaired visual processing, particularly affecting visual acuity and contrast sensitivity (Chung et al., 2017).

Moreover, the expression levels of key molecular markers such as C-Fos protein, GABA, and BDNF significantly reduced in neurons receiving input from the contralateral, deprived eye compared with those receiving input from the ipsilateral eye in MD kittens (Li et al., 2021; Yu, 2014; Cheng et al., 2008). Additionally, recent evidence indicates that high concentrations of magnesium can promote recovery of binocular visual function in amblyopia, an effect mediated by transient receptor potential melastatin-like 7 (TRPM7) channels, further highlighting potential molecular targets for amblyopia treatment (Dai et al., 2024). Activity-regulated cytoskeleton-associated protein (ARC/Arg3.1), induced in neurons by neural activity, plays an essential role in activity-dependent synaptic plasticity and is also critical for memory consolidation and cognitive flexibility (Barylko et al., 2018). Recent studies demonstrated that MD increases neuronal dysfunction in LGN, accompanied by a significant reduction of ARC/Arg3.1 expression, whose changes from visual deprivation-driving may contribute to synaptic impairment and subsequent amblyopia development (Fan et al., 2023).

**FIGURE 1:**
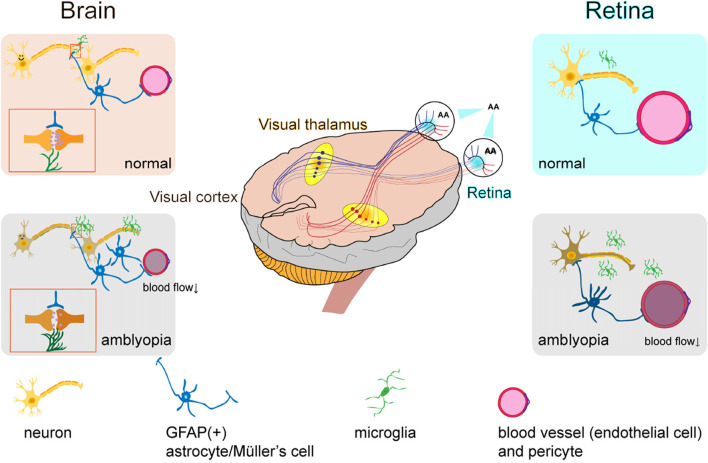
Hypothesized neurovascular unit alterations in the retina and brain of amblyopic individuals.

These findings suggest that multiple neurotransmitter systems and their downstream signaling pathways play multifaceted roles in regulating NVU function in amblyopia. As our understanding of these molecular mechanisms deepens, they are emerging as highly promising therapeutic targets, particularly for interventions aimed at restoring visual function beyond the traditional critical period. Based on the above literature review, we propose the following hypothesis regarding NVU alterations in the retina and brain of individuals with amblyopia (Figure 1), referencing mechanisms described in pathological myopia and diabetic retinopathy.

In the retina, blood flow parameters may be altered, characterized by reduced flow velocity and vascular narrowing, which leads to retinal malnutrition. This results in impaired local neurovascular coupling, functional changes in neurons, dysfunction of pericytes and endothelial cells, activation of microglia, and a more stellate morphology of GFAP-positive structures such as astrocytes and Müller cells. In the brain, especially in the primary visual cortex (V1), cerebral blood flow is significantly reduced, neuronal responsiveness is diminished, internodal segments of myelin become shorter, astrocyte numbers increase, and microglia are activated.

## 5 Future directions

In amblyopia, neurons, blood vessels, and glial cells undergo parallel and interdependent alterations that collectively shape the structural and functional plasticity of the visual system—from the retina to the brain. Imbalanced visual input first disrupts neuronal activity, leading to impaired synaptic efficacy, reduced cortical responsiveness, and long-term deficits in plasticity. These changes are accompanied by decreased cerebral blood flow and disrupted neurovascular coupling (NVC), which impair the metabolic support of active neural circuits and exacerbate functional deterioration. At the cerebral level, mismatches between neuronal activation and blood flow have been observed in V1, the lateral geniculate nucleus (LGN), and frontoparietal associative pathways, indicating that NVC dysfunction is a core pathological feature. At the retinal level, amblyopia models demonstrate structural remodeling of the NVU, including altered ganglion cell function, reduced capillary density, changes in vessel caliber, and impaired perfusion. In parallel, glial elements such as astrocytes and Müller cells show morphological and activation changes under visual deprivation, suggesting their involvement in neurovascular signaling and metabolic maintenance.

Together, these findings highlight amblyopia as a multi-layered disorder involving systemic dysregulation of the neuro-glia-vascular unit, rather than a purely cortical dysfunction. Future therapeutic approaches should aim to restore this integrated unit across both retinal and cortical circuits, offering novel molecular and cellular targets to promote visual recovery even beyond the traditional critical period.

Amblyopia has traditionally been studied primarily from a neuronal dysfunction perspective; however, recent evidence focusing on the NVU reveals a more complex pathogenesis involving synergistic dysregulation among neurons, glial cells, and the vascular network. By integrating clinical findings and animal model data, this review highlights how dysfunction within the NVU, including neurons, glia, and vasculature, extends beyond the classical neuronal-centric view of amblyopia. However, there are still too few studies on the fusion of amblyopia and neurovascular units, compared with diabetic retinopathy, etc. New insights advocate for multi-target interventions directed toward the NVU, potentially overcoming limitations associated with conventional single-target therapies.

Developing high spatiotemporal resolution *in vivo* imaging technologies, such as the integration of adaptive optics and two-photon microscopy, will be critical for capturing real-time interactions among neurons, glia, and vasculature during the critical period of amblyopia. Combining optogenetic manipulation with organoid models enables precise interrogation of specific neurovascular unit components involved in the closure of visual plasticity windows. The integration of visual organoids with multimodal imaging platforms allows for multiscale, high-resolution investigation of NVU pathology in both the retina and visual cortex during amblyopia, offering key support for cross-species mechanistic alignment and clinical translation. Existing studies have demonstrated that retinal and brain organoids can form structurally and functionally connected systems. When combined with optical imaging, functional electrophysiology, and single-cell omics, these organoid-based platforms provide a highly controllable and human-relevant model for exploring NVU interactions and therapeutic strategies in amblyopia (Fligor et al., 2021). This approach not only reduces the reliance on animal models but also offers an irreplaceable advantage by closely recapitulating human pathological features.

Protein and lipid profile, spatial transcriptomics and single-cell metabolomics could be used to characterize molecular profiles of each NVU component at distinct developmental stages of amblyopia, emphasizing spatiotemporal changes in glial cell polarization phenotypes (e.g., astrocyte A1/A2 conversion). These techniques have been applied to other diseases to explain pathological changes (Yang et al., 2023; Li et al., 2021), which could be convincing reference to amblyopia. Special attention should be given to paracrine signaling and glial-endothelial interactions, focusing on the dynamic alterations in vascular-glial crosstalk mediated by endothelial, astrocytic, and pericytic factors.

Developing multi-target approaches to modulate NVU dysfunction may be useful for the diseases with multiple retinal-brain interactions. Exploring neurovascular-coupled hydrogel scaffolds designed to emulate the mechano-chemical microenvironment of the NVU, thereby promoting visual pathway remodeling through enhanced neurovascular coupling might be a new way. Additionally, integrating brain-computer interface (BCI) technology with local vasomotor signal monitoring may establish novel “neurovascular bidirectional feedback” treatments, enhancing therapeutic precision through real-time neuromodulation and vascular response adjustments.

Addressing translational gaps by examining the reversibility of NVU impairments in adult amblyopia, potentially overcoming the current age-related therapeutic constraints. Identifying clinically relevant biomarkers using integrated neuroimaging and multi-omics approaches could guide personalized interventions. Moreover, exploring neurovascular effects of established therapies (e.g., levodopa, fluoxetine) may clarify their role in NVU modulation. Defining sensitive biological markers for NVU dysfunction, such as retinal and cerebrovascular complexity measures or inflammatory cytokine profiles, offers promising opportunities for personalized medicine and targeted interventions beyond conventional age limitations.

Finally, provided that strict ethical approval, informed consent, tissue preservation, and standardized treatment protocols are rigorously adhered to, it is feasible to obtain human tissue samples from certified biobanks or *postmortem* donor banks. Increasingly, this approach is recognized as a valuable method for research. Such samples offer unique and irreplaceable insights into human-specific neurovascular and glial mechanisms underlying amblyopia, particularly when integrated with transcriptomic and histological analyses. However, the availability of samples from pediatric populations, especially those affected by amblyopia, remains severely limited. Consequently, non-human species continue to play a critical role in experimental modeling. Although rodents possess certain anatomical limitations—such as the absence of a fovea—they are well suited for mechanistic and genetic investigations. In contrast, cats and non-human primates exhibit greater anatomical and functional homology to the human visual system, notably in aspects of binocular integration and cortical plasticity. Therefore, a complementary approach is warranted, integrating bioinformatics, multi-species models, biobank-derived sample data, *in vitro* platforms, and advanced human imaging techniques. This integrated strategy aims to mitigate the limitations inherent to individual model systems and enhance translational relevance.

In conclusion, the review have combed the role of NVU changes in the brain and retina for clinical and experimental animal studies, hoping to provide ideas in the mechanisms and treatments of amblyopia.
